# When the Good Outweighs the Bad: Day‐Level Interactions Between Positive and Negative Alcohol Consequences and Their Impact on Next‐Day Alcohol Cognitions

**DOI:** 10.1111/acer.70427

**Published:** 2026-10-06

**Authors:** Scott E. King, Jack T. Waddell, William R. Corbin

**Affiliations:** ^1^ Department of Psychiatry University of Washington Seattle Washington USA; ^2^ Department of Psychology Arizona State University Tempe Arizona USA

**Keywords:** alcohol consequences, drinking evaluations, ecological momentary assessment, young adults

## Abstract

**Background:**

Alcohol consequences are among the most studied predictors of future drinking behavior, yet findings regarding the influence of negative consequences on subsequent alcohol use remain inconsistent. It is possible that the more frequent experience of positive consequences, which are more salient to drinking behavior, outweighs the impact of negative consequences. The current study examined how both domains of alcohol consequences and their co‐occurrence influenced next‐day evaluations of drinking episodes and subsequent drinking behavior among young adults.

**Method:**

Eighty‐four young adults (Mage = 22.73, SD = 1.54; 56% female at birth) enrolled in an 18‐day ecological momentary assessment study. Across 1412 days (578 drinking days), participants completed morning reports assessing prior‐day drinking behavior, prior‐day positive and negative consequences, and evaluations of the drinking episode. Multilevel models tested indirect associations between alcohol consequences and next‐day drinking via subjective evaluations, as well as the interaction between concurrent positive and negative consequences.

**Results:**

Experiencing more positive consequences than usual was associated with more favorable evaluations of the drinking episode, including higher overall positivity ratings (*b* = 0.23, SE = 0.03, *p* < 0.001) and greater perceived worth (*b* = 0.16, SE = 0.02, *p* < 0.001), whereas greater‐than‐usual negative consequences predicted less favorable positivity ratings (*b* = −0.24, SE = 0.05, *p* < 0.001) and lower worth ratings (*b* = −0.16, SE = 0.05, *p* < 0.001). Positive consequences moderated the relation between negative consequences and overall evaluations (*b* = 0.06, SE = 0.03, *p* < 0.05), such that the impact of negative consequences on ratings of positivity was attenuated at higher levels of positive consequences. Although no prediction of the likelihood of next‐day drinking was observed, more favorable evaluations predicted heavier next‐day drinking quantity on drinking days (IRR = 1.22, 95% CI [1.07, 1.38]). Positive consequences also indirectly predicted heavier next‐day drinking quantity via more favorable evaluations (*b* = 0.05, SE = 0.02, *p* < 0.01).

**Conclusions:**

Findings suggest that positive alcohol experiences may buffer the impact of negative consequences, highlighting the importance of subjective evaluations of drinking episodes in shaping subsequent drinking behavior.

## Graphical abstract

This study examines day‐level interactions between positive and negative consequences in the prediction of next‐day evaluations of drinking episodes. Using ecological momentary assessment data, findings from the current study suggest that although the experience of negative consequences dampens how positively drinking episodes are viewed, this relationship is buffered as increasing levels of positive consequences are experienced. These results provide important insight into potential mechanisms through which risky drinking persists despite the frequent experience of harmful outcomes.

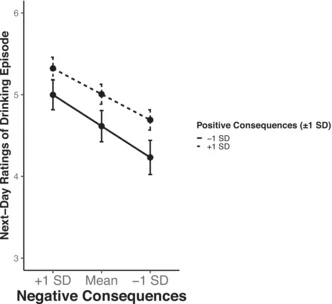

## 1 Introduction

Although recent trends suggest that overall patterns of alcohol use are declining (e.g., Dunphy et al. 2025), alcohol use and misuse remain common and costly among young adults in the United States (e.g., Patrick et al. 2025). Alcohol consequences both positive (e.g., feeling sociable, having a good time; Corbin et al. 2008) and negative (e.g., being hungover, fights/arguments, risky decisions; Read et al. 2006) are some of the most studied constructs in the alcohol literature. Across studies, heavier alcohol use is a robust, strong, and consistent predictor of experiencing negative consequences (e.g., Barnett et al. 2014; Carpenter and Merrill 2021; Merrill, Labhart, and Perks 2023). Unsurprisingly, these findings have been the premise of multiple feedback‐based interventions that seek to lessen the experience of negative consequences while still allowing for the experience of positive, prosocial ones (e.g., Miller et al. 2013; Riper et al. 2009). However, such interventions often rely on feedback regarding past negative consequences despite studies suggesting that negative and positive consequences are highly correlated and often co‐occur.

Social learning theory (Maisto et al. 1999) suggests that the experience of negative consequences should dissuade future drinking whereas the experience of positive consequences should motivate continued use. However, extant literature is mixed regarding the impact of alcohol consequences on future use behavior. Across people, one study found that negative consequences dampened subsequent drinking behavior from week to week (e.g., Barnett et al. 2015), whereas others have found little to no effect both cross‐sectionally and over the span of several years (e.g., Martinez et al. 2014; Read et al. 2013; Waddell et al. 2021). In contrast, prior studies have more consistently demonstrated that, across individuals, positive alcohol consequences are associated with heavier drinking cross‐sectionally and longitudinally (e.g., Park et al. 2013; Patrick et al. 2016). Furthermore, when testing such relations within individuals on a more granular timescale, an equally perplexing pattern emerges. Specifically, research suggests that more frequent positive consequences predict heavier subsequent drinking behavior (e.g., Lee et al. 2018; Zaso et al. 2016), whereas within‐person deviations in negative consequences are often unrelated to next‐day drinking (e.g., Lee et al. 2018; Merrill et al. 2021). Thus, relations between positive consequences and subsequent drinking remain consistent across studies and timescales, yet the impact of negative consequences on future drinking remains unclear.

Most prior studies have focused on explicit use behavior, whereas outcomes such as cognitions may align better with social learning theory's premise that the act of processing information from their experiences informs future behavioral decisions (Neighbors et al. 2013). Despite inconsistent links with future use, research has demonstrated that negative consequences are associated with higher next‐day negative expectancies and lower next‐day willingness to drink (Lee et al. 2018; LoParco et al. 2021), and positive consequences are associated with higher next‐day positive expectancies (Lee et al. 2018). However, additional cognitions outside of expectancies and behavioral willingness may also be sensitive to past‐night drinking experiences and relate to future consumption. In particular, individuals' evaluations of a drinking episode, or the extent to which a drinking experience is viewed positively or negatively overall, may be particularly sensitive to past‐day experiences. This is important as the generally null relations between negative consequences and subsequent drinking behavior may be explained by how the experience of such consequences was evaluated (i.e., was the drinking episode rated as “worth it,” or viewed as a positive overall experience?). Therefore, the first aim of this study sought to understand the distinct impacts of past‐day positive and negative consequences on next‐day drinking evaluations and objective next‐day drinking behavior.

In addition, prior work has mostly considered positive and negative consequences as mutually exclusive constructs rather than considering their potential co‐occurrence. This is notable considering that positive consequences are experienced more frequently than negative consequences (Barnett et al. 2014; Lee et al. 2011; Patrick and Maggs 2008), yet drinking behavior predicts the experience of both (e.g., Merrill et al. 2016; Waddell et al. 2025). Thus, it is possible, and perhaps even likely, for a person to experience both positive (e.g., having a positive social interaction) and negative (e.g., feeling hungover the next day) consequences from a single drinking episode. However, few studies have considered their co‐occurrence. To our knowledge, only two prior studies have simultaneously examined positive and negative consequences, finding that fewer negative and more positive consequences are related to more favorable evaluations of drinking episodes, and subsequent evaluations are influenced by how positively or negatively consequences are viewed (Fairlie et al. 2016; Lee et al. 2011). However, neither study considered the possibility that positive and negative consequences may interact to predict next‐day cognitions. Such unobserved interactions may in part explain the paradoxical findings wherein negative consequences, despite their theoretical influence on subsequent drinking, are insufficient to modify future behaviors. Put differently, it is possible that the influence of negative consequences on next‐day evaluations (and subsequent drinking behavior) is buffered by the presence of positive consequences. If true, such findings could have important implications for adaptive, just‐in‐time interventions that often focus solely on previously experienced negative consequences (Nahum‐Shani and Murphy 2026) as individuals may evaluate the overall drinking episode more positively if pro‐social benefits outweigh deleterious ones. Thus, the second aim of this study is to understand how positive and negative consequences may interact with one another to predict next‐day drinking intentions/plans, evaluations, and willingness to drink.

To address these aims, the current study uses data from an ecological momentary assessment study to examine the impact of positive and negative consequences, and their interaction, on next‐day cognitive and drinking outcomes. Specifically, the current study focused on next‐day drinking behaviors as a primary outcome, while also testing whether next‐day cognitions (i.e., ratings of “worth” and positivity) served as a mechanism through which experienced positive/negative consequences predicted the likelihood (and quantity) of next‐day drinking.

Consistent with Social Learning Theory, it was hypothesized that higher‐than‐average negative consequences on a given drinking day would be associated with less positive next‐day evaluations and a lower likelihood of next‐day drinking, whereas higher‐than‐average positive consequences on a given drinking day would be associated with more positive next‐day evaluations and a higher likelihood of next‐day drinking. As the aims of the current study can be conceptualized as attempting to explain why previous studies have failed to find relations between negative consequences and future drinking, we considered positive consequences as a possible moderator of relations between negative consequences and future drinking. Prior works have also shown that evaluations of negative consequences are less negative in the moment, vary as a function of social and physical contextual effects, and differ if a person is in a more positive mood (Merrill et al. 2018). Therefore, we hypothesized that higher‐than‐average positive consequences (which likely increased positive moods) would moderate relations between higher‐than‐average negative consequences and next‐day evaluations/drinking behavior, such that the impact of negative consequences would become weaker as more positive consequences were reported. Finally, we hypothesized that there would be conditional indirect effects on next‐day drinking via next‐day evaluations/drinking behavior, such that higher‐than‐average negative consequences would indirectly predict less next‐day drinking via less positive evaluations and unplanned drinking, but only when positive consequences experienced were lower‐than‐average.

## 2 Method

### 2.1 Participants

Participants were young adults recruited as part of a larger study on momentary relations between drinking motives, drinking context, and affect. Participants were recruited from across the United States via online advertisements posted to social media websites. Inclusion criteria included being between the ages of 21 and 25, reporting past‐month binge drinking (i.e., 4+ drinks in a night for women and 5+ drinks for men), owning a smartphone, and not currently seeking treatment for alcohol‐related problems. As part of the original parent study, initial eligibility criteria also required reporting at least one solitary drinking episode in the past month, but this criterion was removed early in recruitment due to difficulties enrolling individuals who met this requirement. A total of 94 participants were enrolled in the EMA protocol. Twenty‐six participants were recruited under the original eligibility criteria (i.e., required to report past‐month social and solitary drinking), and the remaining 68 were recruited after the eligibility criteria changed. Thus, the current sample can best be described as oversampling individuals who engage in solitary drinking. The recruitment groups did not differ in terms of age [*F*(1, 92) = 0.82, *p* = 0.37], sex assigned at birth [*F*(1, 92) = 3.74, *p* = 0.06], solitary drinking frequency [*F*(1, 92) = 0.06, *p* = 0.81], past‐month drinking frequency [*F*(1, 91) = 0.01, *p* = 0.92], quantity [*F*(1, 91) = 0.35, *p* = 0.56], nor binge‐drinking frequency [*F*(1, 91) = 0.31, *p* = 0.58].

Eight participants had less than 50% overall compliance and were excluded from analyses. A further two participants did not report any drinking during EMA prompts, resulting in a final analytic sample of *N* = 84. The analytic sample was approximately 56% female assigned at birth and had a mean age of 22.73 (SD = 1.54). The sample was predominantly White (73.8%) followed by Black or African American (11.9%); Asian (9.5%); Hispanic, Latine, or Spanish Origin (7.1%); Native American or Alaska Native, Middle Eastern or North African, and Native Hawaiian or Pacific Islander (all 1.2%). Participants were allowed to select as many identities as applied to them.

### 2.2 Procedures

Online advertisements directed interested participants to an online screening to assess eligibility. Eligible participants were re‐contacted via email and were sent a baseline survey to complete. Upon completion of the baseline survey, participants were scheduled for a video orientation where they were briefed on the study protocol. During the video orientation, participants were instructed to download the EMA phone application TigerAware (Morrison et al. 2018) and were educated about the momentary survey schedule. After completion of the video orientation, participants were enrolled in 18 days of EMA starting the following Thursday after their orientation, allowing for the capture of three weekends. Participants were compensated $45 for the baseline survey and initial zoom orientation, in addition to a maximum of $100 for the EMA protocol, dependent on compliance. On each day of the EMA protocol, participants were sent one morning report at 9 a.m. Due to the aims of the study, data for analyses were restricted to only morning reports in which participants reported prior‐day drinking and consequences.

The following surveys were not included in the data analytic plan and are only outlined below to provide important context for the overall study design. In addition to the morning reports, participants were sent one random afternoon report (between 12 noon and 6 p.m.), and three random nightly reports (6–10:30 p.m.). Participants were instructed to self‐initiate a drink report that was always available to them (i.e., drink initiation reports) if they began drinking, after finishing their first drink. Completion of a drink initiation report then triggered brief follow‐up drink reports every 30 min for the next two‐and‐a‐half hours (i.e., five follow‐up reports), which replaced contemporaneously scheduled reports. Furthermore, if a participant reported drinking in any of the nightly reports, they were redirected to the drink report sequence.

### 2.3 Morning Report Measures

#### 2.3.1 Alcohol Use

Participants were asked to report if they drank alcohol the previous day (0 = no, 1 = yes). If participants reported drinking, they were asked to report how many standard drinks they consumed. Participants were reminded that a standard drink is equivalent to 12 oz. of beer, 5 oz. of wine, or 1.5 oz. of liquor. Reports of drinking from morning reports were reverse lagged onto the previous day to allow for contemporaneous prediction of drinking.

#### 2.3.2 Alcohol Consequences

Each morning participants were asked if they experienced any of 10 negative consequences (ω^within^ = 0.69, ω^between^ = 0.82) and eight positive consequences (ω^within^ = 0.68, ω^between^ = 0.68) (0 = no, 1 = yes), frequently endorsed by young adults (Lee et al. 2017; Merrill, Boyle, et al. 2023; Merrill et al. 2018; see Table 1). Positive and negative consequences were independently summed to create two separate day‐level aggregates representing the total number of positive and negative consequences experienced the night before. Person‐level averages were also computed for positive and negative consequences to account for the average number of consequences experienced per drinking episode.

**TABLE 1:** Item‐level endorsement rates across positive and negative consequence domains.

Please indicate if you experienced any of the following last night as a result of your drinking	Days endorsed (%)	Mean person‐level percent of days endorsed (SD)	Bivariate correlations	
			Rating^a^	Worth^b^
Negative consequences				
Are you hungover right now?^c^	78 (13.5)	14.05% (16.23)	−0.10*	−0.14**
You felt nauseated or vomited	41 (7.1)	7.10% (14.49)	−0.12**	−0.08
You were rude or obnoxious	27 (4.7)	4.27% (9.32)	−0.11*	−0.09*
You hurt or injured yourself by accident	7 (1.2)	1.16% (4.10)	0.01	−0.05
You got into a fight with a friend/family member/significant other	17 (2.9)	2.80% (6.76)	−0.19**	−0.14**
You said or did something while drinking that you regretted	35 (6.1)	5.35% (9.79)	−0.04	−0.03
You forgot parts of the night or things you did	31 (5.4)	5.82% (12.69)	0.08	0.03
You neglected school or work obligations	23 (4.0)	3.95% (10.65)	−0.02	−0.04
You spent too much money on alcohol or food while drinking	61 (10.6)	10.07% (16.77)	0.07	−0.02
You drank more than you planned to	143 (24.7)	23.02% (22.22)	−0.04	−0.12**
Positive consequences				
Things that would normally bother you failed to do so	50 (8.7)	7.67% (13.98)	−0.01	0.05
You were able to express yourself more easily	225 (38.9)	39.71% (28.14)	0.19**	0.14**
You talked to someone you otherwise would not have	114 (19.7)	20.10% (22.99)	0.16**	0.09*
You had a creative moment or experience	92 (15.9)	13.90% (22.44)	0.17**	0.11*
You made a new friend or acquaintance	106 (18.3)	17.27% (19.87)	0.24**	0.13**
You made others laugh	360 (62.3)	61.14% (29.55)	0.23**	0.19**
You had something fun/exciting happen	297 (51.4)	49.13% (28.86)	0.34**	0.23**
You slept better	174 (30.1)	28.37% (32.55)	0.28**	0.25**

^a^ Rating (i.e., how positive/negative the drinking episode was viewed) was scored from 1 = extremely negative to 7 = extremely positive.

^b^ Worth (i.e., Was the drinking episode viewed as worth it?) was scored from 1 = not at all to 5 = very worth it.

^c^ Assessment of hangovers occurred independently of the stem “Please indicate if you experienced any of the following last night as a result of your drinking,” but were treated as a negative consequence. **p < 0.01; *p < 0.05.

#### 2.3.3 Drinking Episode Experience and Ratings

Cognitions related to the previous drinking episode were assessed using two previously used items to examine the impact of negative consequences on subjective evaluations of drinking episodes (e.g., Fairlie et al. 2016; Merrill, Labhart, and Perks 2023). Participants were first asked “Thinking about your overall drinking experience yesterday, how would you rate the experience?” and were asked to rate their experience on a 7‐point scale (1 = extremely negative to 7 = extremely positive). Participants were also asked to respond to the prompt “Thinking about the whole drinking experience yesterday, the positive and the negative, how much was it worth it?” from 1 = not at all to 5 = very worth it. Person‐level averages of rating and worth were both computed to assess average ratings across drinking episodes for each participant. Intraclass correlations for overall ratings of positivity (ICC = 0.32) and worth (ICC = 0.30) indicated sufficient variability across the two levels of analyses to conduct multilevel analyses.

#### 2.3.4 Covariates

##### 2.3.4.1 Demographics

Participants reported their age and sex assigned at birth.

##### 2.3.4.2 Day of week

A dichotomous variable representing weekend versus weekday drinking (0 = weekday, 1 = weekend) was coded such that Thursday–Sunday was considered the weekend (e.g., Piasecki et al. 2014).

### 2.4 Data Analytic Plan

Study hypotheses were tested in Mplus 8.4 via two‐level multilevel models with days (Level 1) nested within individuals (Level 2). Multilevel models tested (1) indirect paths from past‐day positive and negative consequences to next‐day drinking via next‐day evaluations (i.e., rating of experience and evaluation of worth) of the past‐day drinking episode, and (2) examined positive consequences as a moderator of relations between negative consequences and next‐day evaluations in the indirect prediction of next‐day drinking.

Hypotheses were tested via a model building approach. First, an unconditional model was estimated. Specifically, the initial multilevel model estimated direct relations between prior day positive and negative consequences and the overall rating and evaluation of worth of the drinking episode (a‐path), relations between the rating and evaluation of the drinking episode and next‐day drinking (b‐path), and direct relations between positive and negative consequences and next‐day drinking (c‐path). A second, conditional model was then estimated with the inclusion of an interaction term that tested if day‐level positive consequences moderated the a‐path between day‐level negative consequences and next‐day evaluations. Given that study hypotheses were focused on day‐level processes, the interaction between positive and negative consequences in the prediction of next‐day evaluations was specified at Level 1. In the presence of a significant interaction, marginal means and simple slopes were probed at ±1 SD from the mean of positive consequences to better characterize the direction of the observed effect. Conditional indirect effects were tested via the product of coefficients method (Tofighi and MacKinnon 2011).

Level 1 distal outcomes (i.e., likelihood of next‐day drinking and drinking quantity) were modeled via negative binomial hurdle (NBH) models. NBH models allow for the simultaneous prediction of two outcomes; the first being a logistic regression representing the likelihood of any drinking and the second being a truncated negative binomial regression representing the total quantity of drinks consumed (if drinking was reported). The NBH model was specified at the day level (i.e., Level 1), which then included a standard between‐person part (i.e., Level 2) given that the between‐person component of the outcome (i.e., drinking quantity at next event) reflected a person‐level average rather than a count. The hurdle component of the NBH model was exponentiated to reflect the odds ratio (OR) of the next day being a drinking day and the count component was exponentiated to reflect the incidence rate ratio (IRR) of the next‐day drinking quantity (if drinking was reported).

Missing data were handled via full‐information maximum likelihood estimation (MLR) with Monte Carlo Integration with 5000 starts to allow for the estimation of parameters. Day of the week (0 = weekday, 1 = weekend) was included as a Level 1 covariate in both models. Participant age and sex assigned at birth (0 = female, 1 = male) were included as Level 2 covariates. Consistent with recommendations made by Enders and Tofighi (2007), Level 1 continuous predictors (i.e., positive and negative consequences, evaluations of worth, drinking episode rating) were group mean centered (i.e., person mean centered) and Level 2 continuous predictors were grand mean centered (i.e., sample mean centered).

## 3 Results

### 3.1 Descriptive Statistics

Across participants, overall morning report compliance was high (1412 of a possible 1512; 93.39%). A total of 578 drinking days were reported across 84 participants. Most drinking episodes occurred on the weekend (75.78%) and participants reported consuming an average of 4.57 drinks per drinking day (SD = 3.38). Participants reported experiencing an average of 2.45 positive consequences per drinking episode (SD = 1.83) and 0.42 negative consequences (SD = 0.91). Drinking episodes were generally rated as moderately positive (*M*_rating_ = 5.02, SD = 1.03) and worth it (*M*_worth_ = 3.69, SD = 0.88).^1^ Descriptive statistics are reported in Table 2. Between‐ and within‐person bivariate correlations are reported in Table 3.

**TABLE 2:** Descriptive statistics.

Variable	*N*	*M* (SD)	Range
Day‐level descriptives			
Positive consequences	578	2.45 (1.82)	0–8
Negative consequences	578	0.42 (0.91)	0–6
Rating (negative–positive)	577	5.02 (1.03)	1–7
Worth (not worth it–worth it)	577	3.69 (0.88)	1–5
Drinking quantity	578	4.57 (3.37)	0–19
Person‐level descriptives			
Age	84	22.73 (1.54)	21–25
Sex at birth	84	55.95% Female	—
Positive consequences	84	2.38 (1.10)	0–5.45
Negative consequences	84	0.40 (0.64)	0–1.80
Rating (negative–positive)	84	4.96 (0.79)	1–6.54
Worth (not worth it–worth it)	84	3.64 (0.68)	1–5.00
Drinking quantity	84	1.91 (1.82)	0–9.00

*Note:* Day‐level descriptives come from morning reports where participants reported their drinking behavior, consequences, and evaluations from the day prior. Drinking was reported on 578 of the total 1412 study days (40.93% of study days). Variables were reverse‐lagged onto the day prior to allow for the contemporaneous prediction of next‐day drinking from prior day reports.

**TABLE 3:** Bivariate zero‐order correlations.

Variable	1	2	3	4	5	6	7
Within‐person zero‐order correlations							
1. Positive consequences	—	—	—	—	—	—	—
2. Negative consequences	0.33*	—	—	—	—	—	—
3. Ratings of positivity^a^	0.27*	−0.08*	—	—	—	—	—
4. Ratings of worth^b^	0.22*	−0.06	0.71*	—	—	—	—
5. Daily drinking quantity	0.19*	0.29*	0.19*	0.12*	—	—	—
6. Day of the week	0.10*	0.15*	−0.04	−0.07	−0.01	—	—
Between‐person zero‐order correlations							
1. Age	—	—	—	—	—	—	—
2. Sex at birth	0.03	—	—	—	—	—	—
3. Average positive consequences	0.09	−0.28*	—	—	—	—	—
4. Average negative consequences	0.07	−0.08	0.45*	—	—	—	—
5. Average ratings of positivity^a^	−0.03	−0.14	0.46*	0.02	—	—	—
6. Average ratings of worth^b^	−0.07	−0.05	0.30*	−0.12	0.81*	—	—
7. Average drinking quantity	0.02	−0.31*	0.36*	0.27*	0.30*	0.20	—

^a^ Rating (i.e., how positive/negative the drinking episode was viewed) was scored from 1 = extremely negative to 7 = extremely positive.

^b^ Worth (i.e., was the drinking episode viewed as worth it?) was scored from 1 = not at all to 5 = very worth it.

* *p <* 0.05.

### 3.2 Unconditional Model

At the within‐person level, when participants reported experiencing more‐than‐average positive consequences they evaluated their past‐day drinking episode as more positive (*b* = 0.23, SE = 0.03, *p* < 0.001) and more “worth it” (*b* = 0.16, SE = 0.02, *p* < 0.001). In contrast, when participants reported more‐than‐average negative consequences, they reported their past‐day drinking episode as more negative (*b* = −0.24, SE = 0.05, *p* < 0.001) and less “worth it” (*b* = −0.16, SE = 0.05, *p* < 0.001) (Table 4). Positive consequences (OR = 1.08, SE = 0.06, 95% CI = [0.96, 1.21]), negative consequences (OR = 1.22, SE = 0.18, 95% CI = [0.92, 1.63]), ratings of worth (OR = 0.84, SE = 0.11, 95% CI = [0.64, 1.10]), and overall ratings of experiences (OR = 0.88, SE = 0.14, 95% CI = [0.66, 1.16]) were not significantly associated with the likelihood of next‐day drinking. However, drinking episodes in which overall experiences were rated as more positive were associated with heavier next‐day drinking in the count part of the hurdle model (IRR = 1.22, SE = 0.06, 95% CI = [1.07, 1.38]). No other significant relations were found in the count part of the hurdle model (Table 5). Calculation of the indirect effect indicated that positive consequences were significantly associated with heavier next‐day drinking quantity via more positive ratings of the drinking episode (*b* = 0.05, SE = 0.02, *p* < 0.01).

**TABLE 4:** Results from conditional model predicting next‐day evaluations from previous day positive and negative consequences.

Outcome	Predictor	*b*	SE	*p*
Day‐level relations (Level 1)				
WP rating^a^	WP positive consequences	0.21	0.03	< 0.001
	WP negative consequences	−0.50	0.13	< 0.001
	Day of week	−0.11	0.09	0.204
	WP positive consequences × WP negative consequences	0.06	0.03	0.026
WP worth^b^	WP positive consequences	0.16	0.02	< 0.001
	WP negative consequences	−0.24	0.09	0.006
	Day of week	−0.15	0.09	0.105
	WP positive consequences × WP negative consequences	0.02	0.02	0.380
Person‐level relations (Level 2)				
BP rating	BP positive consequences	0.45	0.09	< 0.001
	BP negative consequences	−0.36	0.15	0.016
	Age	−0.04	0.04	0.340
	Sex at birth	0.03	0.12	0.839
BP worth	BP positive consequences	0.31	0.08	< 0.001
	BP negative consequences	−0.43	0.15	0.003
	Age	−0.05	0.04	0.205
	Sex at Birth	0.08	0.13	0.533

Abbreviations: BP, between‐person; SE, standard error; WP, within‐person.

^a^ Rating (i.e., how positive/negative the drinking episode was viewed) was scored from 1 = extremely negative to 7 = extremely positive.

^b^ Worth (i.e., was the drinking episode viewed as worth it?) was scored from 1 = not at all to 5 = very worth it.

**TABLE 5:** Results from conditional hurdle model predicting next‐day drinking.

	Drinking day (Yes/No)					Drinking quantity				
	*b*	SE	*p*	OR	95% CI	*b*	SE	*p*	IRR	95% CI
Within‐person										
Positive consequences	0.078	0.059	0.184	1.081	[0.965, 1.212]	−0.020	0.023	0.383	0.980	[0.936, 1.026]
Negative consequences	0.201	0.145	0.165	1.223	[0.922, 1.625]	0.059	0.052	0.258	1.061	[0.958, 1.175]
Rating	−0.129	0.144	0.369	0.879	[0.663, 1.164]	**0.195**	**0.063**	**0.002**	**1.215**	**[1.074, 1.375]**
Worth	−0.174	0.136	0.199	0.840	[0.644, 1.098]	−0.077	0.068	0.259	0.926	[0.810, 1.060]
Day of week	−0.460	0.233	0.048	0.631	[0.400, 0.995]	0.062	0.117	0.598	1.064	[0.846, 1.338]
Between‐person										
Positive consequences	—	—	—	—	—	0.180	0.210	0.392	—	—
Negative consequences	—	—	—	—	—	**1.007**	**0.505**	**0.046**	—	—
Rating	—	—	—	—	—	0.332	0.460	0.471	—	—
Worth	—	—	—	—	—	−0.092	0.496	0.853	—	—
Age	—	—	—	—	—	0.034	0.125	0.787	—	—
Sex at birth	—	—	—	—	—	−0.480	0.406	0.237	—	—

*Note:* Day‐level predictors of the binary part of the hurdle model are odds ratios, whereas predictors of the continuous part of the model are exponentiated to reflect incident rate ratios. Between‐person predictions of the likelihood of a drinking day were unavailable due to the negative‐binomial hurdle model only being specified at the within‐person level. Sex at birth is coded such that 0 = female, 1 = male. Bolded values represent statistically significant values at *p* < 0.05.

### 3.3 Moderation by Positive Consequences

At the within‐person level, there was a significant interaction between positive and negative consequences when predicting the overall rating of the episode (*b* = 0.06, SE = 0.03, *p* < 0.05) but not in the prediction of how “worth it” participants viewed the drinking episode (*b* = 0.02, SE = 0.02, *p* > 0.05). Post hoc probing of simple slopes suggested that greater negative consequences were associated with less positive ratings of drinking episodes at low (*b* = −0.58, SE = 0.17, *p* < 0.005), mean (*b* = −0.50, SE = 0.13, *p* < 0.001), and high (*b* = −0.41, SE = 0.09, *p* < 0.001) levels of positive consequences (Figure 1), but the magnitude of the effect decreased as positive consequences increased. Thus, at the within‐person level, although the experience of more negative consequences was associated with less positive evaluations of the drinking episode, when more‐than‐average positive experiences were also reported, drinking episodes were viewed more favorably. Conditional indirect effects were also observed such that previous negative consequences indirectly contributed to lower next‐day drinking quantity via less‐positive evaluations of the drinking episode, with a stronger effect at low (−1 SD; *b* = −0.11, SE = 0.06, *p* < 0.05) relative to high (+1 SD; *b* = −0.08, SE = 0.04, *p* < 0.05) levels of positive consequences.

**FIGURE 1:**
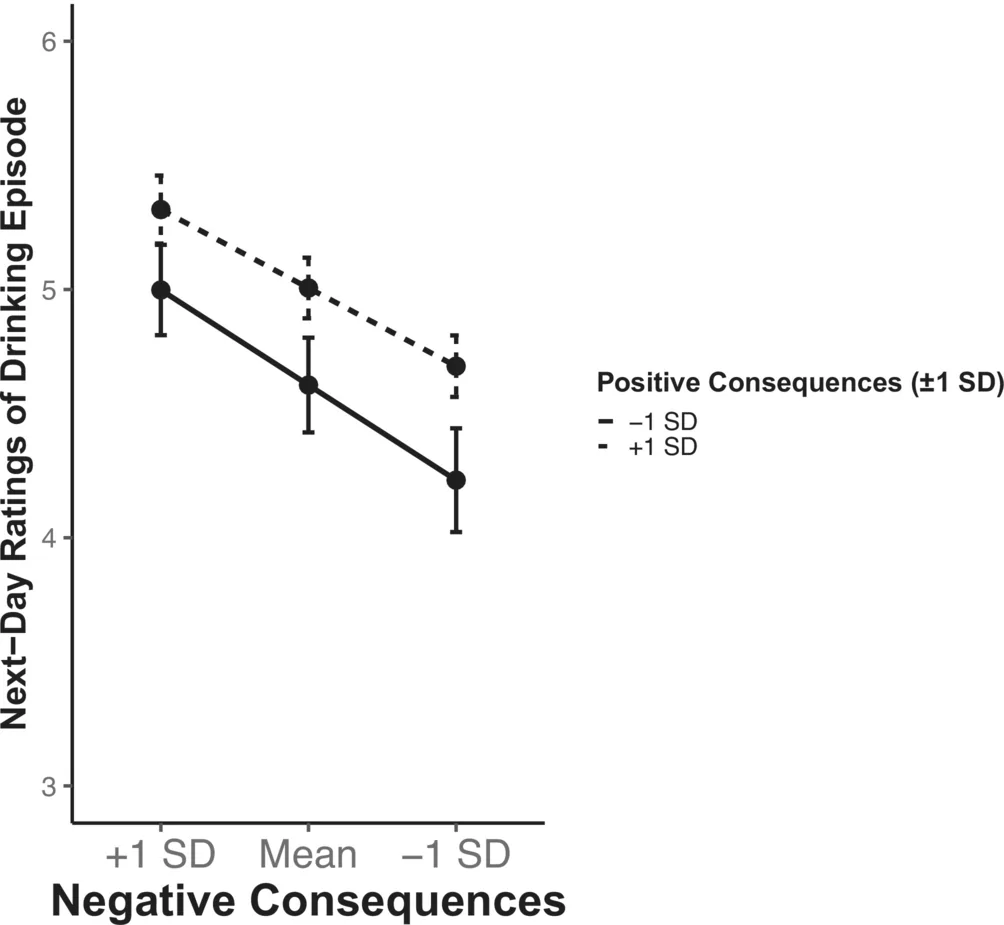
Day‐level interaction between positive and negative consequences in the prediction of next‐day evaluations. Negative consequences decreased how positively drinking episodes were evaluated the next day, but the experience of high (+1 SD) positive consequences significantly buffered that effect relative to low (−1 SD) positive consequence days.

### 3.4 Supplemental Analyses

Given that most drinking episodes occurred on weekends, supplemental analyses were conducted to determine if relations differed when analyses were restricted to drinking episodes that only occurred on the weekend (*N* = 438), thus reducing potential barriers for next‐day drinking. Several differences emerged.

At the within person level, rating of the overall drinking episode was again associated with positive (*b* = 0.23, SE = 0.03, *p* < 0.001) and negative (*b* = −0.48, SE = 0.12, *p* < 0.001) consequences in the expected direction. Although higher‐than‐average positive consequences were still associated with evaluating drinking episodes as more “worth it” (*b* = 0.18, SE = 0.03, *p* < 0.001), negative consequences were no longer significantly related (*b* = −0.17, SE = 0.07, *p* = 0.073). A significant interaction between positive and negative consequences predicting next‐day ratings was also observed (*b* = 0.05, SE = 0.02, *p* = 0.040) such that although negative consequences were associated with less‐favorable ratings at low (−1 SD; *b* = −0.55, SE = 0.15, *p* < 0.001), mean (*b* = −0.48, SE = 0.12, *p* < 0.001) and high (+1 SD; *b* = −0.41, SE = 0.09, *p* < 0.001) levels of negative consequences, the effect was again weaker as positive consequences increased (Table S1).

When looking at prediction of next‐day drinking (and heaviness of drinking), positive consequences emerged as a predictor of the likelihood of next‐day drinking (*b* = 0.14, SE = 0.07, *p* = 0.042, RR = 1.15). No other variables were associated with the likelihood of next‐day drinking. Furthermore, although rating of episode (*b* = 0.19, SE = 0.07, *p* < 0.01) continued to be associated with heaviness of drinking, ratings of worth became inversely related to next‐day drinking quantity (*b* = −0.14, SE = 0.07, *p* < 0.05). Results are reported in Table S2.

## 4 Discussion

Theoretical models of behavior assert that behaviors are more likely to continue when reinforced by positive outcomes and less likely when reinforced by negative ones. However, patterns of alcohol use seem to deviate from this trend wherein individuals often continue to drink at elevated rates despite the presence of negative outcomes. Thus, using data from an ecological momentary assessment study, the current study examined how experiences of positive and negative consequences shape next‐day cognitions and drinking behavior in daily life. Findings indicated that both positive and negative consequences were robustly associated with next‐day evaluations of a drinking episode. Positive consequences also moderated relations between negative consequences and next‐day ratings, such that the effects of negative consequences on next‐day ratings were weaker at higher levels of positive consequences. Finally, positive consequences indirectly predicted greater next‐day drinking quantity (contingent upon drinking) via more positive evaluations. Together, findings highlight the importance of considering both co‐occurring consequences and proximal cognitive processes in understanding drinking behavior at the daily level.

### 4.1 Prediction of Next‐Day Evaluations

Consistent with prior work, positive and negative consequences demonstrated opposing associations with evaluations of drinking episodes (Fairlie et al. 2016; Lee et al. 2011). On days when individuals experienced more positive consequences than usual, they evaluated their drinking episodes as more favorable and more “worth it,” whereas days characterized by more negative consequences were associated with less favorable evaluations. Fairlie et al. (2016) previously found that specific domains of positive (fun/social, image enhancement, relaxation) and negative (social, personal) consequences most strongly influenced overall evaluations of the drinking episode, and that consequences that were experienced as the most favorable/aversive were the most influential on subsequent evaluations. The observed interaction between positive and negative consequences in the current study extends this work by providing additional insight into how these experiences are processed. Specifically, positive consequences buffered the impact of negative consequences on next‐day evaluations, suggesting that, although negative consequences were associated with less favorable evaluations across all levels of positive consequences, their impact was attenuated when drinking episodes were also characterized by more positive outcomes.

Importantly, these findings provide nuance to prevailing theoretical models of behavior, such as social learning theory (Maisto et al. 1999), that suggest that positive and negative consequences have opposing and relatively independent effects on behavior. Rather, the current study suggests that such experiences are not mutually exclusive and are more likely integrated in such a way that negative outcomes are weighed against the concurrent experience of positive consequences. This is notable given that positive consequences are experienced more frequently than negative ones (Barnett et al. 2014; Patrick and Maggs 2008). Furthermore, cognitive theories of decision‐making suggest that individuals rely on heuristics, such as the frequency of experiences or overall affective evaluations, when forming judgments (e.g., Slovic et al. 2007). Therefore, within this framework, repeated pairing of alcohol use with positive outcomes may sensitize individuals to the rewarding properties of drinking. Over time, this process may lead to a bias in which positive outcomes disproportionately influence evaluations and decision‐making, even in the presence of negative consequences.

### 4.2 Prediction of Next‐Day Drinking

Notably, neither positive nor negative consequences, nor evaluations of the drinking episode, were associated with the likelihood of drinking the next day. One potential explanation is that the decision to drink may be more habitual or contextually driven for certain individuals and thereby more influenced by stable routines, social opportunities, or environmental cues than by prior drinking experiences (e.g., Lau‐Barraco et al. 2016). In contrast, positive evaluations of drinking episodes were associated with heavier subsequent drinking, consistent with (a) the idea that higher subjective value strengthens the likelihood of repeating a behavior (Sutton and Barto 2018) and (b) prior research demonstrating that positive alcohol‐related experiences are more reliable predictors of heavier drinking (Park et al. 2013; Patrick et al. 2016).

The differential prediction of next‐day drinking initiation versus the intensity of next‐day drinking supports prior work suggesting that different processes may explain the initiation versus escalation of alcohol use (e.g., Fairlie et al. 2019). Furthermore, the observed indirect pathway from positive consequences to heavier drinking via more positive evaluations suggests that global evaluations of drinking episodes may play a more meaningful role in shaping *how much* individuals drink rather than *whether* they drink at all. Importantly, previous research has suggested that neither the experience of positive nor negative consequences nor the perceptions of these negative consequences influenced subsequent drinking behavior (Merrill et al. 2021). However, the current study suggests that relations between consequences and next‐day drinking may operate via evaluations of the drinking episode itself, rather than evaluations of the consequences. Future studies may benefit from considering how the subjective evaluation of consequences may contribute to overall evaluations of the drinking episode to better characterize day‐level mechanisms of risk for heavy drinking.

Supplemental analyses that restricted data to drinking episodes that only occurred on the weekends largely supported primary findings while also suggesting that different evaluative judgments may exist depending on when drinking occurs. Consistent with primary analyses, positive consequences were associated with more favorable next‐day evaluations and positive consequences again interacted with negative consequences to dampen the effect of negative consequences on next‐day ratings. However, negative consequences were no longer significantly associated with ratings of worth, and ratings of worth were inversely associated with next‐day drinking quantity. This suggests that negative consequences may have a disproportionate effect on future drinking when drinking occurs on the weekday as individuals may have to engage in more in‐depth cognitive reappraisals of their drinking experiences or rationalize their experiences more deeply compared to when they drink on the weekend in a relatively “risk free” context. The inverse relation between ratings of worth and next‐day quantity may also suggest that drinking episodes that are particularly worth it may lead to a level of satiation where motivations to drink may be lower if they received a higher level of satisfaction the night before, and such relations may be more prevalent on weekends (vs. weekdays).

Taken together, the primary and supplemental findings suggest that the impact of negative consequences on evaluations is weakened in the presence of positive outcomes, thus providing a potential explanation into the limited influence negative consequences have on drinking behavior change. Furthermore, a conditional indirect effect in which negative consequences led to less next‐day drinking via more negative evaluations was also observed, although this effect too was buffered by the presence of positive consequences. Collectively, these findings may suggest that negative consequences, if they existed independently of positive consequences, may indeed be enough to reduce future drinking. However, the positive, prosocial benefits of alcohol appear to buffer the immediate impact of negative consequences. In addition, these interactions were observed among a high‐risk sample of young adults who likely experience more negative consequences when compared to their lighter drinking peers. The more frequent experience of these consequences may inoculate more heavy drinkers against the deleterious effects of such experiences, whereas lighter drinkers may have less frequent experiences of negative consequences and therefore be more susceptible to their influence. Future studies may benefit from examination of similar relations in populations with differing patterns of alcohol consumption to better characterize for whom positive consequences may exert the strongest influence.

### 4.3 Future Implications

The current study offers a potential explanation for why negative consequences often do not predict reductions in subsequent drinking (e.g., Martinez et al. 2014; Read et al. 2013; Waddell et al. 2021). This has important clinical implications, as findings suggest that raising awareness of the likelihood of negative consequences, as many momentary interventions are designed to do (e.g., Wright et al. 2017), may be insufficient to facilitate behavior change. In line with harm reduction perspectives (Richards et al. 2021), the findings also suggest that interventions that focus on reducing drinking quantity when individuals are drinking rather than reducing overall drinking frequency may be more effective at reducing alcohol harm. In sum, findings from the current study suggest that, rather than increasing awareness of the likelihood of consequences themselves, future interventions may benefit from increasing the salience or perceived cost of negative consequences, or by encouraging more deliberate, less automatic processing of drinking experiences to reduce the salience of positive outcomes.

### 4.4 Limitations

Study limitations should be considered. First, the sample consisted of young adults who were oversampled to report solitary drinking and reported recent binge drinking, which may limit generalizability to lower risk drinkers. Second, consequences were assessed as counts of endorsed items, which may not capture the subjective severity, subdomains of consequence typologies (e.g., social vs. dependence‐related consequences), or personal significance of these experiences. It is possible that varying positive and negative consequences may carry different weight in shaping evaluations of drinking experiences. For example, a single severe consequence may drive evaluative judgments more than several minor ones, and a single extremely positive consequence may have similar weight relative to more neutral experiences. Future research may benefit from incorporating multidimensional measures of consequences as well as measures of perceived consequence severity to better understand cognitions directly associated with drinking experiences. Furthermore, person‐centered or item‐response approaches that weight consequences by their severity as rated by study participants may also help disentangle the roles frequency, severity, and/or type of consequence play in informing post‐drinking evaluations and decisions to drink again. Finally, although the present study focused on evaluations of drinking episodes, other cognitive processes such as intentions to drink, willingness to experience consequences, and affective experiences while drinking may also play important roles and should be examined in future work.

Despite limitations, the current study demonstrated that positive and negative consequences shape next‐day evaluations of drinking episodes. Importantly, positive consequences attenuated the impact of negative consequences, suggesting that individuals integrate co‐occurring experiences when forming overall appraisals. Although these factors were not associated with the likelihood of next‐day drinking, more favorable evaluations predicted heavier subsequent drinking among those who reported drinking, highlighting their role in shaping consumption intensity. Study findings suggest that alcohol‐related consequences may influence behavior indirectly through cognitive evaluations, underscoring the importance of targeting how individuals weigh positive and negative aspects of their drinking in future intervention and prevention efforts.

## Conflicts of Interest

The authors declare no conflicts of interest.

## Supporting information


**Table S1:** Results from conditional model predicting next‐day evaluations from previous day positive and negative consequences on weekend drinking days.
**Table S2:** Results from conditional hurdle model predicting next‐day drinking on weekend drinking days.

## Data Availability

The data that support the findings of this study are available from the corresponding author upon reasonable request.

## Endnotes

^1^
Rating (i.e., how positive/negative the drinking episode was viewed) was scored from 1 = extremely negative to 7 = extremely positive. Worth (i.e., was the drinking episode viewed as worth it?) was scored from 1 = not at all to 5 = very worth it.
