# Supplementary material for: When the Good Outweighs the Bad: Day‐Level Interactions Between Positive and Negative Alcohol Consequences and Their Impact on Next‐Day Alcohol Cognitions
Source: Alcohol Clin Exp Res (Hoboken). 2026 Oct 6;50(10):e70427. doi: 10.1111/acer.70427 (PMC13643195; doi:10.1111/acer.70427)
Supplement: Supplementary file 1 [file ACER-50-0-s001.docx]

| **Day-Level Relations (Level 1)** | | | | |
| --- | --- | --- | --- | --- |
| Outcome | Predictor | *b* | SE | *p-*value |
| WP Rating^1^ | WP Positive Consequences | 0.23 | 0.03 | < 0.001 |
|  | WP Negative Consequences | -0.48 | 0.12 | < 0.001 |
|  | WP Positive Consequences x WP Negative Consequences | 0.05 | 0.02 | 0.040 |
| WP Worth^2^ | WP Positive Consequences | 0.18 | 0.03 | < 0.001 |
|  | WP Negative Consequences | -0.17 | 0.09 | 0.073 |
|  | WP Positive Consequences x WP Negative Consequences | 0.004 | 0.02 | 0.852 |
| **Person-Level Relations (Level 2)** | | | | |
| Outcome | Predictor | *b* | SE | *p-*value |
| BP Rating | BP Positive Consequences | 0.45 | 0.09 | < 0.001 |
|  | BP Negative Consequences | -0.36 | 0.15 | 0.016 |
|  | Age | -0.04 | 0.04 | 0.340 |
|  | Sex at Birth | 0.03 | 0.12 | 0.839 |
| BP Worth | BP Positive Consequences | 0.31 | 0.08 | < 0.001 |
|  | BP Negative Consequences | -0.43 | 0.15 | 0.003 |
|  | Age | -0.05 | 0.04 | 0.205 |
|  | Sex at Birth | 0.08 | 0.13 | 0.533 |

**Supplementary Table 1. Results from conditional model predicting next-day evaluations from previous day positive and negative consequences on weekend drinking days**

**Note.** Analyses were restricted to only drinking days that occurred on the weekend (i.e., Thursday – Sunday). WP = Within-Person; BP = Between-Person; SE = Standard Error.

Rating (i.e., how positive/negative the drinking episode was viewed) was scored from 1=extremely negative to 7=extremely positive.

^2^ Worth (i.e., Was the drinking episode viewed as worth it?) was scored from 1=not at all to 5=very worth it.

**Supplementary Table 2. Results from conditional hurdle model predicting next-day drinking on weekend drinking days.**

|  | Drinking Day (Yes/No) | | | | | Drinking Quantity | | | | |
| --- | --- | --- | --- | --- | --- | --- | --- | --- | --- | --- |
|  | *b* | SE | *p* | OR | 95% CI | *b* | *SE* | *p* | IRR | 95% CI |
| **Within-Person** |  |  |  |  |  |  |  |  |  |  |
| *Positive Consequences* | 0.078 | 0.059 | 0.184 | 1.081 | [0.965, 1.212] | -0.020 | 0.023 | 0.383 | 0.980 | [0.936, 1.026] |
| *Negative Consequences* | 0.201 | 0.145 | 0.165 | 1.223 | [0.922, 1.625] | 0.059 | 0.052 | 0.258 | 1.061 | [0.958, 1.175] |
| *Rating* | -0.129 | 0.144 | 0.369 | 0.879 | [0.663, 1.164] | **0.195** | **0.063** | **0.002** | **1.215** | **[1.074, 1.375]** |
| *Worth* | -0.174 | 0.136 | 0.199 | 0.840 | [0.644, 1.098] | -0.077 | 0.068 | 0.259 | 0.926 | [0.810, 1.060] |
| *Day of Week* | -0.460 | 0.233 | 0.048 | 0.631 | [0.400, 0.995] | 0.062 | 0.117 | 0.598 | 1.064 | [0.846, 1.338] |
| **Between-Person** |  |  |  |  |  |  |  |  |  |  |
| *Positive Consequences* | -- | -- | -- | -- | -- | 0.180 | 0.210 | 0.392 | --- | --- |
| *Negative Consequences* | -- | -- | -- | -- | -- | **1.007** | **0.505** | **0.046** | --- | --- |
| *Rating* | -- | -- | -- | -- | -- | 0.332 | 0.460 | 0.471 | --- | --- |
| *Worth* | -- | -- | -- | -- | -- | -0.092 | 0.496 | 0.853 | --- | --- |
| *Age* | -- | -- | -- | -- | -- | 0.034 | 0.125 | 0.787 | --- | --- |
| *Sex at Birth* | -- | -- | -- | -- | -- | -0.480 | 0.406 | 0.237 | --- | --- |

**Note.** Analyses were restricted to only examining relations on drinking days that were reported on the weekends (i.e., Thursday-Sunday). Day-level predictors of the binary part of the hurdle model are odds ratios, whereas predictors of the continuous part of the model are exponentiated to reflect incident rate ratios. Between-person predictions of the likelihood of a drinking day were unavailable due to the negative-binomial hurdle model only being specified at the within-person level. Sex at birth is coded such that 0 = female, 1 = male.
